# Sensory Nudges: The Influences of Environmental Contexts on Consumers’ Sensory Perception, Emotional Responses, and Behaviors toward Foods and Beverages

**DOI:** 10.3390/foods9040509

**Published:** 2020-04-17

**Authors:** Han-Seok Seo

**Affiliations:** Department of Food Science, University of Arkansas, 2650 North Young Avenue, Fayetteville, AR 72704, USA; hanseok@uark.edu; Tel.: +1-479-575-4778; Fax: +1-479-575-6936

**Keywords:** nudge, sensory, context, consumer behavior, perception, acceptability, emotional response

## Abstract

Food products with highly acceptable flavors are not always successful in the marketplace. Sales of identical food products sold in two different stores often differ. Patrons’ choices of specific menu items vary depending on menu designs at restaurants. Such examples suggest that consumer behavior related to eating, preparing, or purchasing foods and beverages is typically complex, dynamic, and sensitive. There is a growing body of evidence that environmental cues surrounding foods and beverages can modulate consumer perception and behavior in the context of eating and drinking. In light of increasing interest in environmental cues, this Special Issue was designed to introduce recent research that highlights how sensory cues derived from environmental cues can modulate consumer perceptions, emotional responses, and behavior related to foods and beverages. The eleven articles addressed in this Special Issue provide informative and insightful findings that may be applied to a wide range of food-related sites, including grocery stores, retail markets, restaurants, dining facilities, and public dining areas. The findings from these articles also suggest that product developers, sensory professionals, retailers, marketers, and business owners should consider not only sensory aspects of food products, but also sensory cues derived from surrounding contexts to better understand consumer perception, acceptability, and behavior toward their food products.

## 1. Introduction

Numerous factors can lead to difficulties in predicting consumer perception, preference, or behavior toward foods and beverages. For example, consumer acceptability of coffee beverages has been found to differ as a function of (1) coffee-related variables (e.g., coffee variety, processing condition, or sensory attribute profile [1,2]); (2) consumer-related variables (e.g., demographical, physiological, or genetic variations [3,4]); and (3) environment-related variables (e.g., ambient condition, situational condition, packaging condition, or cup/container type [5,6,7]; for a review, see [8]). In a similar vein, Meiselman [9] highlighted that three principal contextual variables: the food itself, the individual, and the eating situation, influence individual acceptance and consumption of foods and beverages.

Breeders, food processors, and food scientists have expended much effort toward improvement of food quality and increase in consumer acceptability [10]. Sensory evaluation techniques have also been used to characterize sensory profiles of food products and ensure their consumer acceptability before their market introduction. Interestingly, although consumer acceptance testing conducted prior to market introduction had shown that consumer panelists rated the target products highly acceptable, low success rates of the products in food businesses were often reported [11]. Therefore, to better understand variations in consumer acceptability and behavior related to food choice and consumption, more attention has been paid to roles of other non-sensory factors such as individual variations and environment-related contexts. There has, in particular, been growing interest in determining the effects of environmental contexts on the modulation of consumer perception, acceptability, food choice, and food intake (for a review, see [12,13,14]). More specifically, consumer perception, acceptance, purchase-related behavior, and food consumption-related parameters have been found to vary with numerous contextual factors, including tableware/container condition [6,7,15], condiment availability [16], ambient background sound [17,18], ambient scent type [19,20,21], ambient temperature level [22], and labeling information [23,24,25]. Moreover, in recent years, immersive technologies were able to facilitate researchers in manipulating environment-related variables to test how contextual variables might affect consumer perception, acceptability, and behavior toward foods and beverages [26,27]. 

“Nudges” are small contextual cues aimed at gently pushing people to judge and behave toward achieving specific goals that can be helpful in improving health, social welfare, sustainability, or happiness in our society [23,28,29]. In fact, the term “nudge,” defined as “*any aspect of the choice architecture that alters people’s behavior in a predictable way without forbidding any options or significantly changing their economic incentives*” (p. 6), was originally introduced by Thaler and Sunstein [28] in the behavioral economics field. The concept of nudging has been adapted and applied to a wide range of fields, including nutritional science [23], politics and public policy [30], and public health [31]. Nudge-related interventions must be easy and cheap to implement, non-mandatory, and helpful in leading to positive decisions and behavior [28], and nudge interventions have been classified in a diverse way (for a review, see [32,33]).

Environmental context-related sensory cues derived from either unimodal or multimodal systems, such as ambient scent and/or background music, can play the role of nudges (“choice architectures”) that aim to lead consumers to choose or eat foods considered helpful for achieving healthy, balanced, or sustainable diets [34] (for a review, see [35]). In the broader sense, the concept and application of sensory nudges described in this Special Issue may be extended to sensory-related contextual cues designed for modulating not only food intake or food choice/purchase-related behavior, but also consumer perception of, or emotional responses to, target food or beverage items. As mentioned above, an increasing number of studies have provided empirical evidence that consumer perception and acceptance of, even emotional responses to, foods or beverages can differ with environmental contexts, i.e., sensory cues surrounding the target foods or beverage items [7,18,36]. In light of a growing body of evidence revealing the effect of sensory nudging in the field of sensory and consumer sciences, this Special Issue was designed to introduce original research and systematic reviews contributing to a deeper understanding of how sensory-related contexts affect consumers’ sensory and emotional response, food intake, and food choice/purchase-related behavior. This Special Issue includes nine original research articles and two systematic review articles representing 37 authors with 20 different affiliations in six countries. It is of particular interest that contextual cues related to a variety of sensory modules, including visual, olfactory, auditory, somatosensory, and multisensory systems, were introduced as sensory nudges.

## 2. Nudging of Sensory-Related Contextual Cues

### 2.1. Visual Cues

Visual cues of environmental contexts (e.g., colors, sizes, shapes, or display patterns) have been popularly used for modulating consumer perceptions and behaviors in the contexts of food consumption and food choice/purchase [23] (for a review, see [14]). For example, while yellow lighting was found to increase participants’ appetites, blue and/or red lighting decreased their willingness to eat [37,38]. As another example, strawberry mousse served on a white plate tasted significantly sweeter than the same mousse served on a black plate [36]. Earlier studies also showed the influence of container weight on food perception, expected satiety, and willingness to pay [39,40]. Building on previous research associated with the nudging effect of visual or touch cues, Mielby et al. [41] in their study combined color cues with weight cues of receptacles (cups). More specifically, the authors conducted a study to test how both cues could affect liking and perception of three differently-flavored carbonated beverages presented in four different types of receptacles that varied in color (red or black) and weight (lighter or heavier). Participants were found to perceive stronger carbonation when they tasted flavored carbonated beverages served in red receptacles than when they were served in black receptacles. Although no significant effects of receptacle weight on liking and attribute intensities (sweetness, sourness, bitterness, and carbonation) were observed, participants perceived more carbonation when the carbonated beverages were presented in heavier receptacles than in lighter receptacles. Notably, a receptacle weight-induced increase in carbonation was found only when beverages were also perceived to be highly bitter, suggesting a complex interaction between intrinsic (bitterness) and extrinsic (weight) cues with respect to carbonation perception.

While transparent packaging designs permitting consumers to see either entire or partial portions of the products have become increasingly popular [42,43], little is known about their effect on consumer perception and behavior in the contexts of foods and beverages. Simmonds et al. [44] designed an online study with the purpose of determining how the positional features of a transparent window that enables consumers to directly see the product depicted on the packaging could influence product evaluation. The authors placed the transparent window at six different positions: top, bottom, top-right, top-left, bottom-right, and bottom-left on the packaging of four different categories of product (granola, chocolate, lemon mousse, and pasta). The study showed that, across all categories, participants would evaluate a product as more positive and attractive when the transparent window was placed on the right side rather than on the left side of the packaging. In particular, granola and pasta products were evaluated as more attractive when the transparent window was located at the packaging bottom rather than at the packaging top. These results suggest that packaging designers, food processors, marketers, and sensory professionals should seriously consider how to optimize size, shape, and position of such transparent windows for improving consumer experiences with food products. 

Motoki et al. [45] raised an interesting question as to whether and how one could infer that someone would be more likely to prefer sweet foods based on her/his facial shape, i.e., round-, neutral-, or angular-faced. A series of three sub-studies found that participants were able to infer that round-faced individuals, more than neutral- or angular-faced individuals, would prefer sweet foods, and the inference was found to be mediated by the thought that obese individuals are more likely to have round faces and prefer sweet foods. Although obese individuals do not always exhibit round faces, and there are also cultural variations in facial shapes, their findings provide practical information that might be applied as a marketing tool in retail stores and food-service areas. For example, a combination of facial recognition techniques and artificial intelligence-related data science may better predict individual consumer preferences for food products or perhaps even non-food products, thereby personalizing menus and advertisements directed toward them [46].

Increasing visibility of target products through visual images or visible spatial arrangement is a key factor at the point of purchase [47,48,49]. Coucke et al. [50] implemented visual nudges in an in-store environment with the purpose of testing combined effects of visual cues related to product display in a real supermarket, i.e., a size of product-display area and a quantity of displayed product, with respect to sales of poultry meat. In that study, the authors clearly showed that enhancing the visibility of poultry meat by increasing both types of product display cues resulted in an increase in the amount of poultry meat sold. When the enhanced visibility was subsequently reduced to the previous condition, the amount of poultry meat sold decreased, validating the effect of visual nudges on the product sale. Taken together, the result from their study strengthens the notion that greater visibility results in enhanced sales in the store environment.

The number of item options offered for consumer choice was found to play an important role in affecting consumer acceptability and satisfaction. More specifically, consumers are often more satisfied with their choice when they could select an item from some optimum number of item options rather than from too few or too many options [51,52]. Onuma and Sakai [53] conducted an interesting study aimed at determining whether consumers would evaluate their self-chosen food items more palatable than the food item given. Participants were first asked to choose a tea bag from among three, nine, or twelve options, then asked to taste both the tea item that they had selected and another tea item the experimenter suggested, in a sequential monadic fashion. In fact, although the two tea samples were identical, the result showed that participant rated the tea sample chosen by them from nine options as more palatable than the sample provided by the experimenter only. Such differences were not observed when the number of options was either three or twelve. In other words, self-choice from a manageable number of options (e.g., nine options in this study) increased consumer acceptability of food or beverage items, while a self-choice from a non-optimum (i.e., too small or too large) number of options did not affect consumer acceptability. It should be also noted that since the optimum number of item options may vary with item type, food business owners, retailers, and marketers should carefully explore the number of choices offered before they display options for consumer choice at their stores, on their menus, or at online market sites.

There has been a surge of recent interest in using immersive technologies such as virtual reality, augmented reality, mixed reality, or simulated immersion, to both increase consumer engagement level and capture contextual influences on consumer perception and behavior to food products [26,27,54]. In this Special Issue, Picket and Dando [55] implemented custom-recorded 360-degree videos and their corresponding sounds using a virtual reality (VR) headset to create two different immersive contexts: a typical college bar and a tasting room at a local winery. To better understand the effects of congruent immersive contexts on consumer perception, liking, and willingness to pay toward alcoholic beverages, the authors asked participants to consume and evaluate beer and wine samples in the two different virtual contexts. In their study, participants both liked a wine sample more and were willing to pay more for it when they consumed it in the virtual winery context. Although the effects of congruent virtual context were not observed for the other drink (beer), this study provides a hint as to how utilizing VR technology to explore whether such simulated contexts might serve as a sensory nudge in a realistic setting. 

### 2.2. Auditory Cues

A growing body of studies suggests that background sounds, whether musical piece, noise, or dialogues, can alter consumer perception, liking, and behavior toward foods and beverages [17,18,56,57,58]. Lin et al. [59] conducted a study aimed at determining whether consumer perception of, and emotional responses to, chocolate gelato samples could be affected by environmental sounds varying in affective dimensions (valence, arousal, and dominance) and psychoacoustical characteristics (e.g., roughness and sharpness). Using the temporal check-all-that-apply (TCATA) method, participants selected all attributes perceived from the same gelato sample over a period of 45 s in the presence of five different recorded sounds: at a café, a fast food restaurant, a bar, a food court, and a park. The gelato sample was also consumed under a silent control condition. The results showed that flavor perception of identical gelato samples differed as a function of affective dimensions and psychoacoustical characteristics of environmental sounds. Bitterness, cocoa flavor, and roasted flavor attributes were found to be more related to gelato samples consumed under unpleasant and arousing sound conditions (e.g., bar or fast food restaurant sounds), while sweetness and creaminess attributes were more associated with gelato samples consumed under more pleasant sound conditions (e.g., park or café sound). The results from their study will be helpful for food-service professionals willing to optimize ambient sound conditions for improving consumer experiences of target foods and beverages in their business areas. Furthermore, the findings draw attention to the need for sensory professionals to track temporal variations with respect to background sound-induced perception and acceptability of foods and beverages.

### 2.3. Olfactory Cues

Ambient scents have been shown to affect patrons’ dining experiences, including perceived quality of meal items, dining pleasure, and money spent at the restaurant [19,20]. Since patrons often interact with wait staffs during their dining, it could be also interesting to explore the effects of scents emanating from restaurant wait staffs on patrons’ dining experiences. Singh et al. [60] designed a study aimed at determining whether olfactory cues (body odors) from restaurant wait staff could influence patrons’ dining experiences and interpersonal behavior in a mock restaurant setting. Patrons were asked to choose and consume one of four chicken-meat menu items (baked, broiled, fried, and smoked chicken) in the presence of one of the most likely scents of wait staff: congruent (smoky barbecue scent), fragrance (perfume scent), and no scent (control), applied to fabric aprons of wait staff. The results showed that female patrons gave not only higher ratings of overall liking and satisfaction with respect to meal items, but also gave larger tips to wait staff under fragrance scent conditions compared to those under the no-scent condition. However, such effects of wait-staff scents were not observed in male patrons. Furthermore, patrons consumed chicken menu items under the congruent scent condition significantly less than under the fragrance-scent or the no-scent condition. The results from that study suggest that optimizing wait-staff scents should be positively considered because certain scents can enhance patrons’ dining experiences, a potential advantage in the highly-competitive restaurant industry. 

While people typically consume foods or beverages during a series of multiple bites or chews, participants in many sensory studies of foods or beverages were likely to rate intensities or likings after only an initial bite or sip. Recent studies have shown that sensory evaluation using multiple bite/sip assessments resulted in better performance in terms of product description, variation in temporal dominance of sensation, identification of specific sensations, and determination of overall liking or desire to eat [61,62,63]. Gotow et al. [64] conducted a study to determine whether perceptual sensitivity of retronasal odors could change over a series of sips of an oolong tea beverage. Using multi-sip time intensity (TI) analysis, participants rated perceived intensities of retronasal odors for 60 s after swallowing a sip of oolong tea. The results showed that four TI parameters: (1) maximum intensity (I_max_), (2) time point at which intensity reached maximum value (T_max_), (3) area under the TI curve (AUC), and (4) the rate of intensity increase between the first time points with values exceeding 5% and 90% of the maximum intensity (R_inc_), significantly differed among the first and subsequent nine trials of sipping. More specifically, while the I_max_, AUC, and R_inc_ values were significantly lower for the first trial than for subsequent trials, the T_max_ values behaved the other way around. In other words, the first sip of oolong tea led participants to perceive retronasal odors of oolong tea less intensely, requiring more time to perceive the maximum intensity, than did the subsequent sips. The decrease of retronasal odor intensity was also faster in the second through fourth trials than in the first and the fifth through tenth trials of sips. Taken together, the results emphasize not only that first biting/sipping, but also multiple biting/sipping should be considered when determining consumer perception and liking of food and beverage products. This finding will help product developers and sensory professionals better capture the dynamic characteristics of their products.

### 2.4. Touch Cues

In food and beverage contexts, most studies dealing with touch cues have focused on the effect of oral touch cues (e.g., mouth-feel) on consumer perception and liking of foods and beverages [65,66]. While consumers often experience food and beverage products using their hands during point-of-sale transactions, product usage, and product consumption [7,67], little attention has been paid to the influences of hand-feel touch cues on consumer perception, acceptance, and behavior toward the products. In this Special Issue, Pramudya and Seo [68] systematically reviewed hand-feel touch cues and their influences on consumer perception, acceptability, and experiences with foods and beverages. They first specified key concepts and terminologies related to sense of touch and addressed anatomy and physiology of that sense. Second, the authors divided numerous factors influencing hand-feel touch perception into three categories: product-related (e.g., sensory attributes of product), consumer-related (e.g., demographic, physiological, and psychological factors), and external interface-related factors (e.g., packaging, container, tableware, and cutlery items). Third, the authors reviewed the effects of hand-feel touch cues on perceptions from other sensory modules, i.e., visual, auditory, olfactory, gustatory, and oral somatosensory senses. Fourth, they addressed previous studies that had shown the effects of hand-feel touch cues on consumer emotions and purchase-related behaviors. Finally, the authors suggested multiple ways to apply hand-feel touch cues in the food and beverage industries. This review will help guide food processors, packaging designers, sensory scientists, and marketers in incorporating hand-feel touch cues into their products, thereby upgrading consumer experiences and satisfaction with their target products. 

### 2.5. Multisensory Cues

In this Special Issue, Spence [69] contributed to a systematic review of a concept of “complexity” and its applications in the preparation of a meal item and in the meal item itself. Although the term complexity appears to be conceptual and can be variously interpreted depending on the areas and items applied [70,71], complexity is generally perceived to be a desirable attribute by consumers’ food and drink experience [71,72]. Complexity is also more likely to be related to dynamic, multiple, holistic, and time-spanning than static, single, analytical, and momentary characteristics. Spence reviewed previous literature and practical examples used in real dining settings and food industries with respect to two main aspects. First, Spence approached how the complexity concept has been used for production and preparation of meal items and drinks, including menu engineering, recipes, the number of elements in a dish, the number of courses in a dining menu, and mixing/blending of elements in the drinks. Second, he reviewed multiple key factors in a meal related to the complexity concept with respect to the number of molecules, mixture levels of molecules, and temporal evolution and changes in elements and flavor/mouth-feel perception. This review provides a better understanding of the concept of complexity and its current and potential applications into culinary, dining, and food industries, increasing popularity of the complexity concept in the context of food and beverage businesses. 

To summarize, a total of eleven articles introduce the effects of sensory-related contextual cues on consumer perception, acceptability, and behavior with respect to foods and beverages from different perspectives. While further studies should be conducted to determine how the nudging effects of sensory cues may vary as a function of demographic variables, cultural background, and product type, the findings from these articles provide substantial insights into how to utilize sensory cues during eating, preparing, and selling food and beverage products.

## References

[1] Geel L., Kinnear M., de Kock H.L. (2005). Relating consumer preferences to sensory attributes of instant coffee. Food Qual. Pref..

[2] Sunarharum W.B., Williams D., Smyth H.E. (2014). Complexity of coffee flavor: A compositional and sensory perspectives. Food Res. Int..

[3] Masi C., Dinnella C., Monteleone R., Prescott J. (2015). The impact of individual variations in taste sensitivity on coffee perceptions and preferences. Physiol. Behav..

[4] Ufer D., Lin W., Ortega D.L. (2019). Personality traits and preferences for specialty coffee: Results from a coffee shop field experiment. Food Res. Int..

[5] Kim S.-E., Lee S.M., Kim K.-O. (2015). Consumer acceptability of coffee as affected by situational conditions and involvement. Food Qual. Pref..

[6] Carvalho F.M., Spence C. (2019). Cup colour influences consumers’ expectations and experience on tasting specialty coffee. Food Qual. Pref..

[7] Pramudya R.C., Choudhury D., Zou M., Seo H.-S. (2020). “Bitter Touch”: Cross-modal associations between hand-feel touch and gustatory cues in the context of coffee consumption experience. Food Qual. Pref..

[8] Spence C., Carvalho F.M. (2020). The coffee drinking experience: Product extrinsic (atmospheric) influences on taste and choice. Food Qual. Pref..

[9] Meiselman H.L., Meiselman H.L., MacFie H. (1996). The contextual basis for food acceptance, food choice, and food intake: The food, the situation and the individual. Food Choice, Acceptance and Consumption.

[10] Monsón F., Sañudo C., Sierra I. (2005). Influence of breed and ageing time on the sensory meat quality and consumer acceptability in intensively reared beef. Meat Sci..

[11] Watson E. Can Big Food Companies Launch Successful New Products? Bernstein Rates 10 Key Launches. https://www.foodnavigator-usa.com/Article/2019/01/12/Can-big-food-companies-launch-successful-new-products-Bernstein-rates-10-key-launches?utm_source=copyright&utm_medium=OnSite&utm_campaign=copyright.

[12] Stroebele N., De Castro J.M. (2004). Effect of ambience on food intake and food choice. Nutrition.

[13] Asioli D., Aschemann-Witzel J., Caputo V., Vecchio R., Annunziata A., Næs T., Varela P. (2017). Making sense of the “clean label” trends: A review of consumer food choice behavior and discussion of industry implications. Food Res. Int..

[14] Spence C. (2018). Background colour & its impact on food perception & behaviour. Food Qual. Pref..

[15] Piqueras-Fiszman B., Spence C. (2012). The weight of the container influences expected satiety, perceived density, and subsequent expected fullness. Appetite.

[16] Pellegrino R., Frederick B., Tijare V., da Silveira Venzel A., Rios A., Gomes T., Santos J., Seo H.-S. (2017). The influence of condiment availability on cuisine selection. Br. Food J..

[17] Spence C. (2012). Auditory contributions to flavor perception and feeding behaviour. Physiol. Behav..

[18] Kantono K., Hamid N., Shepherd D., Lin Y.H.T., Skiredj S., Carr B.T. (2019). Emotional and electrophysiological measures correlate to flavor perception in the presence of music. Physiol. Behav..

[19] Guéguen N., Petr C. (2006). Odors and consumer behavior in a restaurant. Int. J. Hosp. Manag..

[20] Ouyang Y., Bhenke C., Almanza B., Ghiselli R. (2018). The influence of food aromas on restaurant consumer emotions, perceptions, and purchases. J. Hosp. Market. Manag..

[21] Biswas D., Szocs C. (2019). The smell of healthy choices: Cross-modal sensory compensation effects of ambient scent on food purchases. J. Market. Res..

[22] Motoki K., Saito T., Nouchi R., Kawashima R., Sugiura M. (2018). The paradox of warmth: Ambient warm temperature decreases preference for savory foods. Food Qual. Pref..

[23] Cioffi C.E., Levitsky D.A., Pacanowski C.R., Bertz F. (2015). A nudge in a healthy direction. The effect of nutrition labels on food purchasing behaviors in university dining facilities. Appetite.

[24] Samant S.S., Seo H.-S. (2016). Quality perception and acceptability of chicken breast meat labeled with sustainability claims vary as a function of consumers’ label-understanding level. Food Qual. Pref..

[25] Samant S.S., Seo H.-S. (2020). Influences of sensory attribute intensity, emotional responses, and non-sensory factors on purchase intent toward mixed-vegetable juice products under informed tasting condition. Food Res. Int..

[26] Bangcuyo R.C., Smith K.J., Zumach J.L., Pierce A.M., Guttman G.A., Simons C.T. (2015). The use of immersive technologies to improve consumer testing: The role of ecological validity, context and engagement in evaluating coffee. Food Qual. Pref..

[27] Sinesio F., Saba A., Peparaio M., Civitelli E.S., Paoletti F., Monteta E. (2018). Capturing consumer perception of vegetable freshness in a simulated real-life taste situation. Food Res. Int..

[28] Thaler R.H., Sunstein C.R. (2008). Nudge: Improving Decisions about Health, Wealth, and Happiness.

[29] Karlsen R., Andersen A. (2019). Recommendations with a nudge. Technologies.

[30] Hausman D.M., Welch B. (2010). Debate: To nudge or not to nudge. J. Polit. Philos..

[31] Li M., Chapman G.B. (2013). Nudge to health: Harnessing decision research to promote health behavior. Soc. Personal. Psychol. Compass..

[32] Blumenthal-Barby J.S., Burroughs H. (2012). Seeking better health care outcomes: The ethics of using the “Nudge”. Am. J. Bioeth..

[33] Wilson A.L., Buckley E., Buckley J.D., Bogomolova S. (2016). Nudging healthier food and beverage choices through salience and priming. Evidence from a systematic review. Food Qual. Pref..

[34] Tijssen I., Zandstra E.H., de Graaf C., Jager G. (2017). Why a ‘light’ product package should not be light blue: Effects of package colour on perceived healthiness and attractiveness of sugar- and fat-reduced products. Food Qual. Pref..

[35] McCrickerd K., Forde C.G. (2016). Sensory influences on food intake control: Moving beyond palatability. Obes. Rev..

[36] Piqueras-Fiszman B., Alcaide J., Roura E., Spence C. (2012). Is it the plate or is it the food? Assessing the influence of the color (black or white) and shape of the plate on the perception of the food placed on it. Food Qual. Pref..

[37] Suk H.-J., Park G.-L., Kim Y. (2012). Bon Appétit! An investigation about the best and worst color combinations of lighting and food. J. Lit. Art Stud..

[38] Cho S., Han A., Taylor M.H., Huck A.C., Mishler A.M., Mattal K.L., Barker C., Seo H.-S. (2015). Blue lighting decreases the amount of food consumed in men, but not in women. Appetite.

[39] García-Segovia P., Harrington R.J., Seo H.-S. (2015). Influences of table setting and eating location on food acceptance and intake. Food Qual. Pref..

[40] Kampfer K., Leischnig A., Ivens B.S., Spence C. (2017). Touch-flavor transference: Assessing the effect of packaging weight on gustatory evaluations, desire for food and beverages, and willingness to pay. PLoS ONE.

[41] Mielby L.A., Wang Q.J., Jensen S., Bertelsen A.S., Kidmose U., Spence C., Byrne D.V. (2018). See, feel, taste: The influence of receptacle colour and weight on the evaluation of flavoured carbonated Beverages. Foods.

[42] Simmonds G., Spence C. (2017). Thinking inside the box: How seeing products on, or through, the packaging influences consumer perceptions and purchase behaviour. Food Qual. Pref..

[43] Simmonds G., Woods A.T., Spence C. (2019). ‘Shaping perceptions’: Exploring how the shape of transparent windows in packaging designs affect product evaluation. Food Qual. Pref..

[44] Simmonds G., Woods A.T., Spence C. (2018). “Seeing What’s Left”: The effect of position of transparent windows on product evaluation. Foods.

[45] Motoki K., Saito T., Nouchi R., Kawashima R., Sugiura M. (2019). Round faces are associated with sweet foods: The role of crossmodal correspondence in social perception. Foods.

[46] Davenport T., Guha A., Grewal D., Bressgott T. (2020). How artificial intelligence will change the future of marketing. J. Acad. Market. Sci..

[47] Moisander J., Markkula A., Eräranta K. (2010). Construction of consumer choice in the market: Challenges for environmental policy. Int. J. Consum. Stud..

[48] Eisend M. (2014). Shelf space elasticity: A meta-analysis. J. Retail..

[49] Cárdenas M.K., Benziger C.P., Pillay T.D., Miranda J.J. (2015). The effect of changes in visibility and price on fruit purchasing at a university cafeteria in Lima, Peru. Public Health Nutr..

[50] Coucke N., Vermeir I., Slabbinck H., Van Kerckhove A. (2019). Show me more! The influence of visibility on sustainable food choices. Foods.

[51] Lyengar S.S., Lepper M.R. (2000). When choice is demotivating: Can one desire too much of a good thing?. J. Personal. Soc. Psychol..

[52] Hafner R.J., White M.P., Handley S.J. (2018). The Goldilocks placebo effect: Placebo effects are stronger when people select a treatment from an optimal number of choices. Am. J. Psychol..

[53] Onuma T., Sakai N. (2019). Choosing from an optimal number of options makes curry and tea more palatable. Foods.

[54] Liu R., Hannum M., Simons C.T. (2019). Using immersive technologies to explore the effects of congruent and incongruent contextual cues on context recall, product evaluation time, and preference and liking during consumer hedonic testing. Food Res. Int..

[55] Picket B., Dando R. (2019). Environmental immersion’s influence on hedonics, perceived appropriateness, and willingness to pay in alcoholic beverages. Foods.

[56] Muniz R., Harrington R.J., Ogbeidea G.-C., Seo H.-S. (2017). The role of sound congruency on ethnic menu item selection and price expectations. Int. J. Hospit. Tour. Admin..

[57] Wang Q.J., Keller S., Spence C. (2017). Sounds spicy: Enhancing the evaluation of piquancy by means of a customized crossmodally congruent soundtrack. Food Qual. Pref..

[58] Fiegel A., Childress A., Beekman T.L., Seo H.-S. (2019). Variations in food acceptability with respect to pitch, tempo, and volume levels of background music. Multisens. Res..

[59] Lin Y.H.T., Hamid N., Shepherd D., Kantono K., Spence C. (2019). Environmental sounds influence the multisensory perception of chocolate gelati. Foods.

[60] Singh A., Beekman T.L., Seo H.-S. (2019). Olfactory cues of restaurant wait staff modulate patrons’ dining experiences and behavior. Foods.

[61] Antúnez L., Giménez A., Alcaire F., Vidal L., Ares G. (2017). Consumer perception of salt-reduced breads: Comparison of single and two-bites evaluation. Food Res. Int..

[62] Van Bommel R., Stieger M., Boelee N., Schlich P., Jager G. (2019). From first to late bite: Temporal dynamics of sensory and hedonic perceptions using a multiple-intake approach. Food Qual. Pref..

[63] Seo H.-S., Adams S.H., Howard L.H., Brownmiller C., Hogan V., Chen J.-R., Pramudya R.C. (2020). Children’s liking and wanting of foods vary over multiple bites/sips of consumption: A case study of foods containing wild blueberry powder in the amounts targeted to deliver bioactive phytonutrients for children. Food Res. Int..

[64] Gotow N., Omata T., Uchida M., Matsuzaki N., Takata S., Hagiwara I., Kobayakawa T. (2018). Multi-sip time–intensity evaluation of retronasal aroma after swallowing oolong tea beverage. Foods.

[65] Wilkinson C., Dijksterhuis G.B., Minekus M. (2000). From food structure to texture. Trends Food Sci. Technol..

[66] Foster K.D., Grigor J.M.V., Cheong J.N., Yoo M.J.Y., Bronlund J.E., Morgenstern M.P. (2011). The role of oral processing in dynamic sensory perception. J. Food Sci..

[67] Wang Q.J., Spence C. (2018). A smooth wine? Haptic influences on wine evaluation. Int. J. Gastron. Food Sci..

[68] Pramudya R.C., Seo H.-S. (2019). Hand-feel touch cues and their influences on consumer perception and behavior with respect to food products: A review. Foods.

[69] Spence C. (2018). Complexity on the menu and in the meal. Foods.

[70] Spence C., Wang Q.J. (2018). On the meaning(s) of perceived complexity in the chemical senses. Chem. Senses.

[71] Spence C., Wang Q.J. (2018). What does the term ‘complexity’ mean in the world of wine?. Int. J. Gastron. Food Sci..

[72] Parr W.V. (2015). Unraveling the nature of perceived complexity in wine. Pract. Winer. Vineyard.

